# Photosystem I-LHCII megacomplexes respond to high light and aging in plants

**DOI:** 10.1007/s11120-017-0447-y

**Published:** 2017-10-03

**Authors:** Eliezer M. Schwarz, Stephanie Tietz, John E. Froehlich

**Affiliations:** 1 Department of Energy Plant Research Laboratory, Michigan State University, East Lansing, MI 48824 USA; 2 Department of Biochemistry and Molecular Biology, Michigan State University, East Lansing, MI 48824 USA

**Keywords:** Photosystem I, Photosystem II, LHC, Complex, Supercomplex, Megacomplex, High light, Senescence, PsaH, Native gel, Green gel

## Abstract

Photosystem II is known to be a highly dynamic multi-protein complex that participates in a variety of regulatory and repair processes. In contrast, photosystem I (PSI) has, until quite recently, been thought of as relatively static. We report the discovery of plant PSI-LHCII megacomplexes containing multiple LHCII trimers per PSI reaction center. These PSI-LHCII megacomplexes respond rapidly to changes in light intensity, as visualized by native gel electrophoresis. PSI-LHCII megacomplex formation was found to require thylakoid stacking, and to depend upon growth light intensity and leaf age. These factors were, in turn, correlated with changes in PSI/PSII ratios and, intriguingly, PSI-LHCII megacomplex dynamics appeared to depend upon PSII core phosphorylation. These findings suggest new functions for PSI and a new level of regulation involving specialized subpopulations of photosystem I which have profound implications for current models of thylakoid dynamics.

**Electronic supplementary material:**

The online version of this article (doi:10.1007/s11120-017-0447-y) contains supplementary material, which is available to authorized users.

## Introduction

The light reactions of photosynthesis are dynamically regulated in response to changing environmental conditions. This regulation is crucial for efficient energy utilization and to avoid or mitigate photo-oxidative damage (Yamori 2016). Understanding how this is accomplished remains one of the most active areas of research in the field of photosynthesis, as it holds promise for needed improvements in both food and fuel production (Zhu et al. 2010; Long et al. 2015; Ort et al. 2015; Slattery et al. 2015). The light reactions take place in pigmented protein complexes embedded in the thylakoid membranes of the chloroplast.

The thylakoid membranes in higher plants are highly structured, and are traditionally described as being separated into two major components: stacks of disk-shaped membranes, the grana, which are connected laterally by sheets of stroma-exposed membrane, the stromal lamellae. This structuring of the thylakoids produces a compartmentalization of the components of the light reactions, with PSII and the major light-harvesting antenna confined to the appressed regions of the granal stacks, while the bulkier PSI and ATP synthase are excluded to the stromal lamellae and granal end membranes (Andersson and Anderson 1980; Pribil et al. 2014). Although the thylakoid membranes lend structure to the photosynthetic components, it is the protein complexes themselves which create and define the compartments in which they are embedded. The dynamics of the photosystem complexes, and that of the thylakoid structures in which they operate, are fundamental to the regulation of photosynthesis. Indeed, new components and previously underappreciated details of these dynamics are continually emerging.

The sub-compartmentalization of the thylakoids by stacking of the grana comes with apparent kinetic costs, requiring diffusion of components (e.g., plastoquinone, plastocyanin, xanthophylls) to and from far-flung sites of activity in their various redox cycles (Trissl and Wilhelm 1993). Hence, lateral segregation is presumed to confer significant advantages to overcome this inefficiency. As a result, separation of the photosystems is currently believed to be necessary to limit excitonic “spillover” from photosystem II to photosystem I (Haferkamp et al. 2010); however, a full understanding of thylakoid compartmentalization is still lacking. Furthermore, stacking of the grana is variable, and the factors affecting this dynamic morphology are only now becoming better understood (Chow et al. 2005; Anderson et al. 2008; Fristedt et al. 2009). The major light-harvesting antenna, LHCII, is known to be mainly responsible for stacking of the thylakoids, i.e., membranes devoid of other photosystem components still undergo extensive stacking, while membranes lacking LHCII do not stack (Mullet and Arntzen 1980; Day et al. 1984). In addition, the newly discovered CURT proteins, which are responsible for the extreme curvature of the membranes at the grana margins, also appear to be necessary for the formation of granal stacks (Armbruster et al. 2013). The degree of granal stacking appears to control, among other things, the edges of grana that form the interface between the granal stacks and the stromal lamellae. These regions have been identified as the site of PSII degradation, and it is also the site at which both PSII and PSI can interact directly with each other (Tikkanen et al. 2008a, b; Puthiyaveetil et al. 2014; Grieco et al. 2015). Consequently, the various domains of the thylakoids can be defined by their specific protein components. The protein composition of these domains is not static, however, and lateral movement of photosynthetic complexes between the thylakoid compartments appears to be a central aspect of their function.

The regulatory importance of the thylakoid dynamics, as implied above, is suggested by the observation that the degree of stacking of the thylakoids is highly variable, with phosphorylation of LHCII and PSII core components contributing to “unstacking” of the grana (Fristedt et al. 2010; Herbstova et al. 2012). LHCII is phosphorylated during State Transitions, which involves the shifting of a portion of the major light-harvesting antenna from PSII to PSI (Larsson et al. 1983). The re-allocation of the light-harvesting antenna to PSI also requires the minor PSI subunits PsaL and PsaH, which together form the docking site for LHCII (Lunde et al. 2000, 2003; Mazor et al. 2015). Phosphorylation of LHCII in State Transitions is catalyzed by the Stn7 kinase (Bellafiore et al. 2005). The kinase is activated at the b6f complex by the binding of reduced plastoquinol to the complex, thereby linking phosphorylation of LHCII to the redox state of the plastoquinone pool (Shapiguzov et al. 2016). State Transitions also appear to be involved in turnover of the D1 protein of PSII, which is rapidly damaged in proportion to light intensity, even under low light conditions (Kim et al. 1993; Sundby et al. 1993). The phosphorylation of LHCII by Stn7 begins the disassembly of photosystem II complexes and also decreases the stacking of the grana membranes (Staehelin and Arntzen 1983). Both of these processes are thought to be important for facilitating the repair cycle of PSII (Kirchhoff 2013, 2014; Yamamoto et al. 2014; Yoshioka-Nishimura 2016), which requires that damaged photocenters be dismantled at the grana margins (Puthiyaveetil et al. 2014). Phosphorylation of PSII core components by a second kinase, Stn8 (Vainonen et al. 2005), appears to be required for efficient mobilization of damaged PSII centers (Tikkanen et al. 2008a, b). How or whether the activities of the Stn7 and Stn8 kinases are coordinated, and therefore how State Transitions and PSII turnover are interrelated, is a question of intense interest.

While State Transitions are thought to be a short-term adjustment to changing light conditions, signaling through the Stn7 kinase also appears to be integral to a longer-term acclimation mechanism called the Long-Term Response (LTR), which is an adjustment of the relative stoichiometries of PSI, PSII, and LHCII (Bonardi et al. 2005; Dietzel et al. 2008; Pesaresi et al. 2009). For example, a shift to lower light intensities results in an increase in LHCII, while a shift to higher light intensities leads to a reduction of LHCII and an increase in photocenters. The stoichiometry of the photosystems relative to one another is variable by species, but ranges from a PSII/PSI ratio of 1:1 to more than 2:1. Interestingly, despite the stoichiometric abundance of PSII relative to PSI, PSI seems nevertheless to be present in large functional excess, since up to 80% of PSI can be eliminated without hampering normal electron flow (Cook and Miles 1990). The reason for this apparent excess of PSI is unknown, although recent findings suggest that multiple subpopulations of PSI distributed in various complexes within the stromal lamellae and grana margins may have unique functions (Suorsa et al. 2015).

While many of the photosynthetic processes (e.g., State Transitions) outlined above have historically been described in terms of spectroscopic phenomena (Williams 1970), another methodology for analyzing the dynamics of the photosynthetic complexes under various environmental conditions is by native gel electrophoresis. Specifically, the chlorophyll-pigmented proteins, i.e., photosystem I and II core complexes and their associated light-harvesting antennae, can be solubilized from the thylakoid membrane, separated, and visualized directly as green bands on a native gel (Allen and Staehelin 1991; Dreyfuss and Thornber 1994). Depending on the solubilization method, thylakoid components can be separated not only into their respective individual bands, e.g., LHCII monomers and trimers, PSII core monomers, or dimers, but also into their various higher-order assemblies, e.g., PSII core dimers with various numbers of LHCII antenna trimers (C_2_S, C_2_S_s,_ C_2_S_2_M, etc.) (Dekker and Boekema 2005). State Transitions, for instance, can therefore be directly visualized by these techniques as the appearance of a band containing PSI with LHCII; this band is absent in the *stn7* mutant which is incapable of State Transitions (Pietrzykowska et al. 2014). Recent advances in developing better native PAGE methodologies and establishing standardized procedures have seen vast improvements in resolving higher-order thylakoid components, the so-called super- and megacomplexes, as well as increased overall popularity of these techniques for general characterization and phenotyping (Jarvi et al. 2011).

The term “megacomplex” is usually used to refer to very large molecular weight assemblies of photosystems found at the top of a native gel. The number of these assemblies seen on a native gel depends on the strength of the solubilization process, but there appear to be many possible such complexes. In most cases, the majority of these are poorly resolved and remain clustered at the top of the gel, even under electrophoretic conditions designed to favor their mobility (Pietrzykowska et al. 2014). The identification and characterization of these complexes is therefore a relatively new and ongoing effort. Most megacomplexes seem to be higher-order photosystem II structures composed of multiple photocenter units with their associated LHCII antennae. This appears to be due to the densely ordered packing of PSII within the grana and the ensuing release of various fragments of these packed arrays upon solubilization (Albanese et al. 2016). In contrast, few higher-order PSI complexes have been described, although notable exceptions are the characterization of cyclic electron flow complexes of PSI with FNR in Chlamydomonas (Iwai et al. 2010) and PSI with Ndh in Arabidopsis (Peng et al. 2008). A recent work by Suorsa et al., which focused specifically on complexes of the stromal lamellae, identified as many as nine megacomplexes, five of which are PSI complexes and two of which are PSII/PSI heterocomplexes (Suorsa et al. 2015). Furthermore, Suorsa et al. presented a model in which State II involves direct association between Photosystem I and Photosystem II at the grana margins, while PSI that is dissociated from PSII may undergo dimer- or trimerization in the lamellae. While the oligomerization state of PSI in higher plants has long been controversial (Kouril et al. 2005), energetic “spillover” between the two photosystems is long established (Chow et al. 1981), and other recent reports have found direct physical and functional interaction between the photosystems (Grieco et al. 2015; Yokono et al. 2015).

Taken together with evidence that PSI is sometimes found associated with a surprisingly large cohort of LHCII (Prakash et al. 2001a, b; Bell et al. 2015), these findings suggest a more dynamic picture of PSI than is captured in the current model of State Transitions. In this paper, we describe the finding of a series of novel and dynamically regulated, high light-inducible PSI-LHCII megacomplexes and attempt to elucidate their regulation and function. The implications of these findings are discussed in terms of Photosystem I as a multifunctional complex with a role in protecting Photosystem II from high light damage.

## Materials and methods

### Plant material and plant growth conditions

The plant species and varieties used for this work are as follows: *Spinacia oleracea, Amaranthus* ‘opopeo,’ *Nicotiana tabacum* ‘Petit Havana,’ *Pisum sativum* ‘Little Marvel,’ and *Arabidopsis thaliana* ‘Columbia.’ Plants were grown in chambers at room temperature (25 °C) at 50% humidity under 16 h of 150 µE/m^2^/s white fluorescent light unless otherwise noted.

### Thylakoid preparation and solubilization

Thylakoid solubilization is based on the method of Allen and Staehelin (1991). Leaf material was dounce homogenized in 50 mM Tris HCl pH 7.4, 10 mM MgCl_2_, 10 mM KCl (TMK buffer) with protease inhibitors (Sigma protease inhibitor cocktail) and filtered through four layers of Miracloth. Membranes were pelleted by centrifugation at 7000×*g* for 10 min at 4 °C and resuspended in TMK buffer. Aliquots were removed for chlorophyll concentration measurement by the method of Porra et al. (Porra et al. 1989) at 4 °C and the volume of each sample was normalized to yield the same total amount of chlorophyll. Membranes were again pelleted by centrifugation at 7000×*g* for 10 min at 4 °C and the supernatants were removed. Membrane pellets were solubilized on ice for 15 min in TMK buffer containing 30% glycerol, 1% β-decyl maltoside, and 1% *n*-octyl glucoside to a chlorophyll concentration of 1 mg/mL. Solubilized thylakoids were then spun a final time at 10,000×*g* at 4 °C for 10 min to pellet starches. Supernatants were loaded directly onto green gels.

### Green gel electrophoresis and electroelution

Native green gel electrophoresis was performed according to the method of Allen and Staehelin (1991). Native gels were cast shortly before use on the day they were used. Gels were poured with an acrylamide gradient of 6–14% at 37.5:1C. Gels lanes were always loaded on an equal chlorophyll basis at 15 µg of chlorophyll per lane. Electroelution was performed using a Bio-Rad electroelution cell with 3 kD molecular weight cutoff dialysis caps. Identical bands from multiple lanes of the same sample were combined for each electroelution. Eluted protein fractions were immediately denatured by boiling in Laemmli (1970) buffer and frozen at − 70 °C for further analysis.

### Western blotting and silver stains

Western blotting was performed according to standard protocols. All antibodies (i.e., PsaD, PsbA, Lhcb1, PsaH, and PsaL) were purchased from Agrisera and were used at approximately 1/1000 dilution, followed by anti-rabbit HRP-conjugated secondary antibody. Blots were developed using Pierce ECL plus chemiluminescent substrate and exposed to X-ray film (RPI™, Research Products International, Corp). Silver staining was performed using Pierce Mass spectrometry compatible silver staining kit according to the manufacturer’s instructions.

### 77 K chlorophyll fluorescence

Low-temperature (77 K) fluorescence emission spectra were recorded with an in-house built spectrofluorimeter in the spectral range of 600–800 nm and an excitation wavelength of 440 nm. Samples were prepared according to Weis (1985) with minor modifications. Particles of leaf disks (*d* = 6 mm) were ground in a cooled mortar with liquid N2 and 0.2 mL frozen ddH2O. Additionally, cooled quartz particles were added to aid homogenization and the resulting powder was then transferred to NMR tubes to be measured.

### Time-correlated single photon counting (TCSPC)

TCSPC was performed at room temperature on excised green gel slices containing the appropriate complex of interest. These gel slices were sandwiched between glass slides for positioning in the laser beam. The time-resolved fluorescence data were acquired using a time-correlated single photon counting (TCPSC) system. The source is a CW passively mode-locked, diode-pumped Nd:YVO_4_ laser (Spectra Physics Vanguard) that produces 2.5 W average power at 355 and 532 nm, with a 80 MHz repetition rate and 13 ps pulses at both wavelengths. The output of this laser excites a cavity-dumped dye laser (Coherent 702-2), which operates at 435 nm producing 5 ps pulses at a repetition rate of 4 MHz (250 ns inter-pulse spacing). Stilbene 420 dye (Exciton) is used to produce the 435 nm light. The excitation pulse from the dye laser is divided, with a fraction of the pulse sent to a reference photodiode (Becker & Hickl PHD-400-N), and the remainder is directed to the sample. Emission is collected using a 40× reflecting microscope objective (Ealing). The emission polarization components parallel (0°) and perpendicular (90°) to the vertically polarized excitation pulse are separated using a polarizing cube beam splitter (Newport). The two polarized channels are detected separately using microchannel plate photomultipliers (Hamamatsu R3809U-50), with subtractive double monochromators (Spectral Products CM-112) for wavelength selection. The detection electronics (Becker & Hickl SPC-132) collect the polarized data separately, yielding *ca*. 30 ps response functions for each channel. Data acquisition, detector bias, and collection wavelength are all controlled using an in-house-written LabVIEW^®^ (National Instruments) program on a PC. Time-resolved fluorescence data were collected at 10 nm intervals between 670 and 740 nm and a total of 10,000 counts were acquired. Data analysis was performed using Microcal Origin.

## Results

### High light responsive megacomplexes are differentially induced in various plant species

**Fig. 1:**
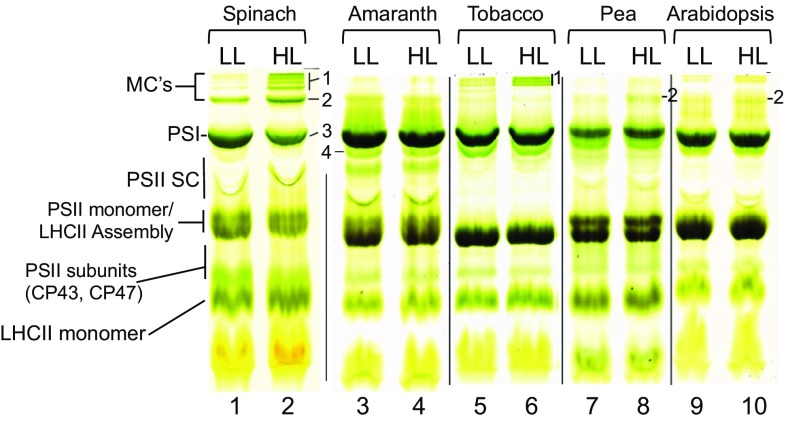
High light-inducible megacomplexes are differentially present in various common model plant species as resolved by native green gel electrophoresis. Species varieties are as follows: *Spinacia oleracea*; Amaranthus ‘opopeo,’ *Nicotiana tabacum* ‘Petit Havana,’ *Pisum sativum* ‘Little Marvel,’ *Arabidopsis thaliana* ‘Columbia.’ Each species was grown under 150 µE/m^2^/s (low light, LL) of illumination and subjected to 600 µE/m^2^/s of white light illumination for 30 min as a high light (HL) treatment. Thylakoids were isolated and solubilized in 1% β-decyl maltoside/1% *n*-octyl glucoside buffer to a final chlorophyll concentration of 1 mg/mL, as described in Materials and Methods, and 20 mg chlorophyll was loaded per lane and analyzed by green gel electrophoresis. Resolved thylakoid complexes designated as megacomplex (MC) bands 1 and 2; Photosystem I (PSI) band 3; PSII dimer band 4 ; Photosystem II Supercomplex (PSII SC); PSII monomer/Light-Harvesting (LHCII) Assembly; PSII Subunits (CP43,CP47); LHCII monomer were labeled according to Allen and Staehlein (1991) and in agreement with our own data from Western blotting

To investigate the dynamic regulation of thylakoid protein complexes, we performed native green gel analysis to survey several common model plant species for response to high light (HL) treatment (Fig. 1). HL treatment for these experiments is defined as plants initially grown under 150 µE/m^2^/s (low light, LL; Fig. 1) of illumination and then transferred to 600 µE/m^2^/s (high light, HL; Fig. 1) of illumination for 30 min. Thylakoid complexes from the various higher plant species were solubilized for native gel electrophoresis with a decyl maltoside/octyl glucoside (DM/OG) detergent system, which has been previously shown by Allen and Staehelin (1991) to resolve thylakoid megacomplexes from Barley, Spinach, Pea, and Arabidopsis, in addition to eight other higher plant and algal species. In addition, the native gel system of Allen and Staehelin (1991) uses low concentrations of the denaturing detergent Lithium Dodecyl Sulfate (LDS) in the running buffer to improve resolution of gel bands. This partially denaturing gel system preferentially destabilizes Photosystem II complexes, allowing cleaner visualization of large PSI complexes. In Fig. 1, using this combination of DM/OG-solubilized thylakoids and green gel electrophoresis, we show the dynamic changes of various photosynthetic complexes in several common model plant species that were transferred from LL to HL conditions. We observed a rapid induction of a cluster of very large complexes at the top of the green gels for several plant species. For the sake of further discussion and clarity we designated this cluster of bands as megacomplexes (MCs), in keeping with established nomenclature for native gel bands in this range (Suorsa et al. 2015). The appearance of these MC bands was most dramatic in spinach, and so the MC bands in spinach were used as a reference for labeling and comparative purposes. The highest molecular weight cluster of bands, of which there were approximately four at the top of our green gel for spinach, we collectively labeled band (1) we also observed a well-defined band that migrated as a separate, single complex, below the cluster of MCs in band 1, which we labeled band (2) finally, we designated the major complex migrating below band 2, as band 3, and the band just below band 3 as band 4 (which was included due to its fluctuation pattern relative to the MC bands, as described below) (Fig. 1).

Intriguingly, we demonstrate in Fig. 1 that different species responded quite dissimilarly when transferred to HL. Specifically, the number of MC bands, as well as the intensity of their induction, was found to differ significantly for different species. The band density in the MC region for Arabidopsis and Pea was especially weak when compared to Spinach. In Tobacco, we observed the induction only of a cluster of MCs at band 1, but not the MC band 2, as was seen for Spinach. Amaranth, in contrast to the other species, seemed to have no response to HL treatment and produced only a series of poorly resolved smaller MC bands that included band 2, but not band 1, and a very strong band 3. The data presented in Fig. 1 demonstrate that the induction of MCs in response to high light treatment varied in plants from species to species. To further investigate the dynamics of MC formation under various environmental conditions, we simplified our experimental approach by selecting spinach as our model plant, since it consistently and conveniently produced an abundance of MCs after transfer to HL.

### High light-inducible spinach megacomplexes are PSI-LHCII complexes and are located in the stromal lamellae

**Fig. 2:**
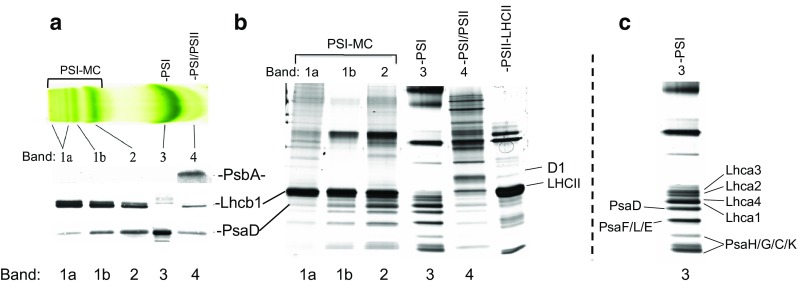
High light-inducible bands are Photosystem I/LHCII megacomplexes. **a**, Western blotting of fractions (i.e., green gel bands 1–4) derived from DM/OG-solubilized spinach thylakoid membranes, probed with antibodies for PsbA, Lhcb1, and PsaD as representative components of Photosystem II (PSII), major light-harvesting antenna (LHCII), and Photosystem I (PSI) complexes, respectively. Bands were excised from gels and proteins were electroeluted so that the components of each band could be compared on a quantitative basis. Normalization was performed on a per chlorophyll basis before samples were denatured and run on standard SDS-PAGE prior to Western blot analysis. **b**, Silver staining of the same samples shown in **a**, but also including the PSII-LHCII supercomplex for comparison. Protein identifications were assigned by overlaying stained gels with Western blots and by comparison with published gels (Jarvi et al. 2011; Scheller et al. 2001). **c** A duplication of Band 3, the main PSI band, from panel **b**, with bands identified by reference to Scheller et al. (2001)

To identify the high light-inducible bands described above, we characterized their protein composition using Western blot analysis (Fig. 2a). For this set of experiments, the cluster of MCs initially designated as band 1 in Fig. 1 was separated into bands 1a and 1b, as shown in Fig. 2a. Bands 1a, 1b, 2, 3, and 4 were first electroeluted from green gels and the proteins recovered were normalized based on chlorophyll content. These samples were then resolved by standard SDS-PAGE and subjected to Western blotting analysis. Figure 2a shows a typical Western blot for bands 1a, 1b, 2, 3, and 4 that have been probed with antibodies to the D1 protein (PsbA), Lhcb1, and PsaD, which were used as representative markers for Photosystem II (PSII), the major light-harvesting antenna (LHCII), and Photosystem I (PSI), respectively. From our Western blot analysis we show that PSII was detected exclusively in band 4, along with PSI and LHCII (Fig. 2a). PSII was not detected in bands 1a,1b, 2, or 3. In contrast, characterization of band 3 detected only PSI, while MC bands 1a, 1b, and 2 all contained both PSI and LHCII. Based on this analysis, Band 3 appears to contain only PSI, while Bands 1a, 1b, and 2 appear to contain PSI associated with LHCII. Intriguingly, Band 4 appears to contain both PSI and PSII. PSI-PSII heterocomplexes have been reported previously in different detergent gel systems from ours (Suorsa et al. 2015). However, it appears likely in this case that the two complexes are simply co-migrating, since the PSII dimer is expected to migrate below PSI at the approximate position of band 4. For the purposes of this work we will refer to this complex simply as the band 4 complex.

In addition to the identification of band components described above, we observed a visible trend of increasing abundance of LHCII relative to PSI as the MCs increased in size (i.e., going from band 3 up to band 1a; Fig. 2a). Likewise, we monitored the trends seen in Fig. 2a by generating a silver-stained gel of the same Bands analyzed in Fig. 2a and subsequently comparing these band profiles with the band profile of the PSII/LHCII band (Fig. 2b). We show that Bands 1a–3 of the silver-stained gel (Fig. 2b) all share a distinct banding pattern on the lower half of the gel that is characteristic of Photosystem I, and which comprises the low molecular weight subunits of PSI, along with the Photosystem I light-harvesting antenna proteins (Lhcas). Figure 2c shows a replicate of Band 3 (i.e., PSI) from Fig. 2b with individual protein bands identified based on reference to Scheller et al. (2001), as well as by overlaying with our own Western blots. In Fig. 2b, one can clearly see the decreasing intensity of staining of PSI small subunits in the MC bands, compared to the PSI main band (Band 3), as LHCII accounts for an increasing percentage of the total chlorophyll in the MCs. We also note that, while staining for the d1 protein is visible in both the band 4 complex and the PSII-LHCII complex in Fig. 2b, there is no detectable staining for D1 in any of MC bands (Fig. 2b). We therefore conclude that the silver staining seen in Fig. 2b confirms the observed trend, seen in Fig. 2a, of PSI associated with increasing amounts of LHCII in the MC bands.

To further verify the trend of increasing amounts of LHCII with increasing MC size, as well as to confirm the identity of the MCs as Photosystem I complexes, the megacomplexes were also normalized by PSI content before Western blotting (Fig. S1). For this normalization, PSI-containing bands 1, 2, and 3 were first excised from green gels and proteins were electroeluted from the gel bands. The samples were then normalized for PSI based on PsaD signal, i.e., samples were loaded so as to produce equivalent signal for PsaD for each band. Blots were then probed for LHCII (Lhcb1) as well as for the additional PSI subunits PsaB, PsaL, and PsaH. As shown in Fig. S1, the trend of increasing LHCII in the MC bands is clear and quite dramatic, with decreased signal for LHCII in band 2, as compared to band1, and no detectable LHCII in the main PSI band, band 3. Interestingly, PsaL and PsaH were also present in dramatically higher ratios, relative to PsaD and PsaB, in the MCs. This trend was even more dramatic for PsaH than for PsaL. Like LHCII, PsaH was undetectable in the main PSI band. This could possibly be due to a loss of PsaH and PsaL during solubilization and electrophoresis, as the two proteins are known to be loosely bound to PSI (Seok et al. 2014; Mazor et al. 2015). These proteins together form the docking site for LHCII on PSI (Lunde et al. 2003). Binding of LHCII to PSI in the MCs may therefore help to stabilize PsaL/H and prevent their loss. Alternatively, these findings may indicate that PsaH and PsaL are not present in all functional PSI complexes in-vivo. Further possible implications of these findings are pursued in the “Discussion” section.

We conclude from the above experiments that the various High Light-induced PSI-MC bands comprise a series of PSI complexes associated with increasing amounts of LHCII. To provide a simple yet comprehensive nomenclature for the remaining complexes resolved by our green gels throughout this paper, we will refer to the spinach megacomplex bands 1 and 2 as PSI-MCs, and band 3 as the main PSI band.

To date, only one PSI-LHCII complex has been identified in the literature, which is the PS-LHCI-LHCII State Transition supercomplex believed to contain only a single LHCII trimer (Bassi and Simpson 1987; Long et al. 2015). The identification of multiple PSI-LHCII complexes of increasing LHCII stoichiometry, as described above, is therefore novel (see Fig. 2a). There are, however, previous reports in the literature of isolation of PSI particles containing surprisingly large amounts of LHCII, i.e., Prakash et al. in senescing cucumber cotyledons (Prakash et al. 2001a, b) and the isolation by Bell et al. of spinach PSI-LHCII complexes containing as many as five LHCII trimers (Bell et al. 2015). Nevertheless, it could be argued that the PSI-MCs described here are simply the result of under solubilization. To test for this possibility, thylakoid samples taken from spinach leaves under growth conditions or after high light treatment were solubilized either normally or at twice the normal detergent to chlorophyll ratio (Fig. S2a). As seen in Fig. S2a, loading half the normal amount of chlorophyll solubilized in the same volume of detergent buffer did not alter the pattern of PSI-MC induction by high light, although some MC density was lost at a higher solubilization strength, as would be expected. We conclude from this experiment that PSI-MC induction is not an artifact of under solubilization.

It could also be argued that the induction of PSI-MCs is not the result of physiological signaling processes taking place in-vivo during high light treatment, but is rather an aggregation effect occurring post-solubilization. To test for this possibility, solubilized thylakoids from moderately high light-treated spinach were allowed to sit on the bench at room temperature for 3 h (Fig. S2b). No increase in PSI-MCs was observed, but instead there was a general decrease in larger complexes, with a concomitant increase in lower weight complexes. This result is the expected indication of native complexes falling apart over time. We therefore conclude that the observed PSI-MCs are not a product of post-solubilization aggregation.

If the PSI-MCs are physiological complexes of PSI and LHCII, as we contend, then they should be located in the stromal lamellae, since PSI is excluded from the grana. Previous studies have shown that treatment of thylakoid membranes with digitonin allows for the selective solubilization of the stromal lamellae, which contains the majority of PSI complexes, as well as trace amounts of the grana margins, which contain both PSI and PSII complexes (Chow et al. 1980). Hence, thylakoids from spinach plants that had been subjected to HL treatment were isolated and solubilized with 1% digitonin to separate the stromal lamellae from grana stacks (Fig. S2c). This treatment resulted in all of the PSI-MC bands being selectively localized to the digitonin-soluble stromal fraction (i.e., stromal lamellae) (Fig. S2c). None of the PSI-MCs localized to the pellet fraction containing the grana. The addition of β-DM to the digitonin-solubilized complexes caused them to break down into separate PSI and LHCII bands (Fig. S2c). We therefore conclude that the PSI-MCs described here are located in the stromal lamellae, as expected for PSI complexes.

Given the localization of the PSI-MCs in the stromal lamellae, we next attempted to determine the average number of LHCII trimers associated with the PSI photocenters. The chlorophyll *a*/*b* ratio of isolated PSI complexes can be used to estimate the number of LHCII antenna trimers associated with PSI, assuming 155 chlorophyll *a* and 19 chlorophyll *b* per PSI center, including all Lhcas, and 24 chlorophyll *a* and 18 chlorophyll *b* per LHCII trimer (Galka et al. 2012). This calculation gives an expected chlorophyll *a*/*b* ratio of 8.16 for PSI-LHCI and 4.84 for the PSI-LHCI-LHCII State Transition supercomplex, for example. Prakash et al. found a chlorophyll *a*/*b* ratio of 2.9 in PSI complexes isolated from senescing cucumber cotyledons (Prakash et al. 2001a, b), while Bell et al. reported an average chlorophyll *a*/*b* ratio of 3.2 for their spinach PSI-LHCII complexes (Bell et al. 2015). In both cases, this indicates approximately three LHCII trimers per PSI, on average. In order to perform a similar analysis, the chlorophyll *a*/*b* ratios for the digitonin-solubilized fractions described above were measured by standard spectrophotometric analysis as described in Materials and Methods. In mature leaves of spinach plants grown at 150 μE/m^2^/s the average chlorophyll *a*/*b* ratio for these complexes was 7.25 ± 1.08, while after high light treatment at 600 μE/m^2^/s the average chlorophyll *a*/*b* ratio was 3.7 ± 0.35. We conclude that under these growth conditions there is approximately one LHCII trimer for every six PSI centers on average under normal growth light, while under high light there is an average of two LHCII trimers per PSI reaction center.

### LHCII in PSI-LHCII megacomplexes is functionally coupled to PSI

While we have demonstrated above that the PSI-MCs appear to be physiological complexes of PSI and LHCII located in the stromal lamellae, it could still be argued that the LHCII is merely associated with PSI, but is not actually functionally coupled to the photocenter. There is already evidence in the literature that higher stoichiometric numbers of LHCII can act as an efficient antenna for PSI. A recent study by Akhtar et al. reports that three LHCII trimers can be energetically coupled to PSI at high efficiency in reconstituted liposomes (Akhtar et al. 2016). Functional coupling of LHCII to PSI centers results in quenching of LHCII fluorescence, while free LHCII trimers have very long-lived fluorescence lifetimes on the order of nanoseconds (Akhtar et al. 2016).

To investigate whether the LHCII in our PSI-MCs is indeed energetically connected to PSI, we first looked qualitatively at PSI-MC fluorescence by visualizing native green gel bands using a standard UV transilluminator (Fig. S3a). As shown in Fig. S3a, there is no visible fluorescence from bands 1, 2, or 3 on the native green gel. PSI-MC fluorescence therefore appears to be highly quenched, which strongly suggests that LHCII in the PSI-MC bands is coupled to PSI.

To further characterize coupling of LHCII to PSI in PSI-MC bands, Time-Correlated Single Photon Counting (TCSPC) was used to determine chlorophyll fluorescence decay lifetimes for the PSI-MC bands 1 and 2, and for the main PSI band. Figure S3b shows typical room temperature fluorescence spectra taken from green gel slices containing the bands to be subjected to TCSPC analysis. Figure S3c shows an overlay of representative raw TCSPC traces at 680 nm for these gel bands, and also includes the LHCII trimer band for comparison. As can clearly be seen in Fig. S3, the fluorescence decay for free LHCII is much slower than that of the other complexes. Figs. S3d–f show stacked Decay Associated Spectra (DAS) for PSI (Band 3), Band 2, and Band 1, respectively. Decay curves were fitted by global analysis for two exponential decays to capture the main spectral components in the tens to hundreds of picoseconds range. The fast decaying component that is normally present for PSI complexes, an approximately 15 ps lifetime at 680 representing the PSI core antenna, is barely resolved by our instrumental setup but is visible at *t* = 0 in Fig. S3d. The spectra for all bands display two prominent peaks at 700 and 720. The increasing complement of LHCII in bands 2 and 3 can be seen to result in shifting of the spectrum towards slower decaying peaks at 680 and 700 (Fig. S3e, f). The lifetimes, reported as an average of at least 3 biological replicates, are as follows: for PSI *τ*1 = 45 ± 9.2 ps and *τ*2 = 330 ± 42.3 ps, for Band 2 *τ*1 = 44 ± 4.7 ps, and *τ*2 = 344 ± 29.2 ps, and for Band 1 *τ*1 = 71 ± 17 ps and *τ*2 = 571 ± 138 ps. These lifetimes are very similar to others reported for PSI-LHCII complexes (Akhtar et al. 2016), although the lifetimes for Band 1 are somewhat longer, presumably due to a larger antenna size. Our findings therefore appear to be consistent with the interpretation that the PSI-MCs described here are functional PSI-LHCII complexes.

### PS-LHCII megacomplexes are formed in response to dark and high light and are modulated by growth light intensity

**Fig. 3:**
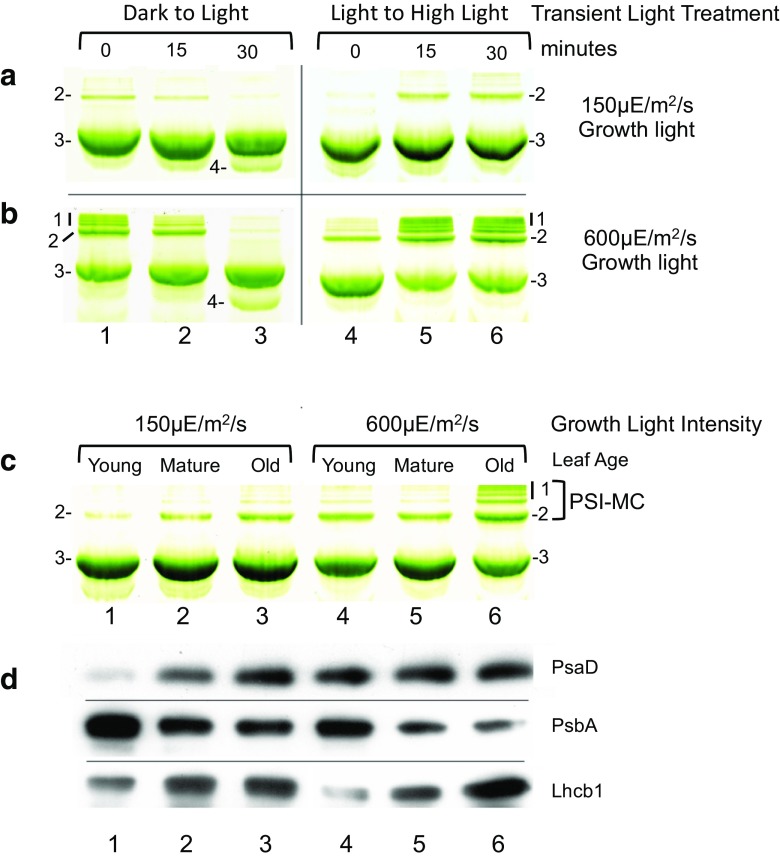
Photosystem I megacomplex induction increases with growth light intensity and leaf age and is correlated with high Photosystem I/II ratios. Growth light intensity dependence of PSI megacomplex induction. Spinach plants were grown under either, **a** moderate (150 µE/m^2^/s) or **b** high (600 µE/m^2^/s) light conditions and the first sets of mature fully expanded leaves were processed for green gel electrophoresis. Dark to Light: leaves were sampled after overnight darkness (0) and 15 and 30 min after the lights were turned on. Light to High Light: Leaves were sampled midday under growth light and after 15 and 30 min of high light treatment. High light treatments were double normal growth light intensity. **c** Interplay between leaf age and growth light intensity on PSI-MC induction. Spinach plants were grown under moderate or high light conditions as in panel **a**. Leaves were grouped into unexpanded (young), fully expanded (mature), or visibly yellowing (old). Leaves of plants grown under high light conditions senesced earlier, so similar leaves were used for comparison rather than identical cohorts. **d** Western blotting of samples derived from green gel shown in panel **c**. A portion of the same solubilized thylakoid samples which had been loaded onto the green gel were denatured in Laemmli buffer and run on standard SDS-PAGE for Western blotting. Samples were loaded on an equal chlorophyll basis

The results presented in Fig. 1 demonstrate that the induction of PSI-MCs is dynamic and appear to be influenced by changing light conditions (i.e., LL transitioning to HL). In order to examine this phenomenon in greater detail, we performed several light-dependent experiments to investigate the conditions under which PSI-MCs (bands 1–2) are induced in spinach thylakoids, as presented in Fig. 3. Chamber-grown spinach plants were used in these experiments to allow control of the light treatments used in our analysis. Spinach plants were grown under either low light intensity (150 μE/m^2^/s) or high light intensity (600 μE/m^2^/s) for 6 weeks and mature leaves were harvested for green gel analysis.

As shown in Fig. 3a, b, lanes 1–3, it was found that PSI-MCs are already present in spinach leaves after an overnight period of darkness, and that these complexes are then rapidly dismantled upon exposure to normal growth light, i.e., with the onset of daylight. Moreover, the intensity and number of PSI-MC bands formed during overnight darkness depended on the intensity of light at which the plants were grown. Plants grown under low light conditions at 150 μE/m^2^/s of illumination accumulated only PSI-MC band two during overnight darkness (Fig. 3a, lane 1). In contrast, plants grown in high light at 600 μE/m^2^/s of illumination accumulated both PSI-MC bands 1 and 2 during overnight darkness (Fig. 3b, lane 1). PSI-MCs were observed to be progressively dismantled as plants transitioned from overnight darkness to growth light, with the dismantling process appearing to be completed within 30 min of exposure to growth light conditions (Fig. 3a, b). Intriguingly, in plants grown under both high and low light intensities, the dismantling of the PSI-MCs can clearly be seen to coincide with the appearance of band 4, i.e., there appears to be a direct tradeoff between the formation of the PSI-MCs and formation of the band 4 complex. Additionally, the amount of band 4 complex that is induced during transition from dark to light was noted to be elevated compared to steady-state levels under normal growth light (In Fig. 3a, b compare lanes 3, 4). The induction of the band 4 complex therefore appears to be a transient phenomenon. We conclude from this set of experiments that exposure to growth light signals dismantling of PSI-MCs and concurrent induction of the band 4 complex.

Growth light intensity was also found to have a profound effect on the extent of PSI-MC formation in response to HL treatment (Fig. 3a, b, lanes 4–6). For HL treatment in this experiment, spinach plants that had been grown at 150 μE/m^2^/s of illumination were transferred to 600 μE/m^2^/s of illumination for 30 min, while plants grown at 600 μE/m^2^/s of illumination were transferred to 1200 μE/m^2^/s of illumination for 30 min. For plants grown at both light intensities the 30 min of HL treatment was initiated at midday, after plants had acclimated to their normal growth light. Plants grown under high light conditions were again found to have greater induction of PSI-MCs than those grown under low light conditions, with induction of both PSI-MC bands 1 and 2 in plants grown at 600 μE/m^2^/s of illumination, while only PSI-MC band 2 was induced in plants grown at 150μE/m^2^/s of illumination. Indeed, as can be seen in Fig. 3a, b, comparing lanes 1–3 with lanes 4–6, the pattern of PSI-MC induction for a given growth light intensity is exactly reversed when going from darkness to growth light, as compared to transfer from growth light to HL treatment. We infer from these experiments that increasing PSI-MC formation is in some way reflective of acclimation to growth at higher light intensities.

### Leaf age and growth light intensity interact to determine PSI-MC induction

During our investigations using chamber-grown spinach plants, we often found that we were not able to observe induction of PSI-MC band 1 in very young spinach plants. We therefore suspected that leaf age might also be contributing to the degree of PS-MC formation. To investigate this possibility, we assayed for the effect of leaf age on PSI-MC induction under both low (150 μE/m^2^/s) and high (600 μE/m^2^/s) growth light intensities (Fig. 3c). Leaves from spinach plants grown continuously under these two light intensities were grouped into young (expanding), mature (fully expanded), and old (visibly yellowing) categories and thylakoids isolated from these leaves were analyzed by green gel electrophoresis. As shown in Fig. 3c, both the density and the number of PSI-MC bands were found to increase with increasing leaf age and growth light intensity. Under low growth light conditions (150 μE/m^2^/s) leaves showed no detectable levels of PSI-MC band 1 at any age, while band 2 was gradually induced with increasing leaf age (Fig. 3c, compare Young to Old). Under high growth light conditions (600 μE/m^2^/s), however, PSI-MC band 2 was already present in young leaves and gradually increased in older leaves. While PSI-MC band 1 was only seen in this experiment in old leaves of plants grown at 600 μE/m^2^/s, additional experiments at higher growth light intensities indicate that PSI-MC band 1 can also be induced even in leaves that are only mature, but not yet old, as the light intensity increases (Fig. S4). Taken together, we conclude from these experiments that leaf age and light intensity act interchangeably and additively to induce PSI-MCs, with band 2 being induced first followed by band 1 as leaf age and/or light intensity increases.

### PSI-MCs are associated with increasing PSI, decreasing PSII

It has previously been shown that long-term changes in growth light conditions and leaf aging have profound effects on the protein composition of the thylakoids, dramatically altering the size of the light-harvesting antenna as well as the ratios of the photocenters to one another (Dietzel et al. 2008). Given these facts, we next looked for possible changes in the major photosystem components that might account for the observed age- and light intensity-dependent induction of PSI-MCs that we observed in Fig. 3c. For this analysis, a portion of the solubilized thylakoid samples used to produce the green gels for Fig. 3c, and which had already been normalized based on total chlorophyll content, were further analyzed by Western blotting (Fig. 3d). PsaD, PsbA (D1 protein), and Lhcb1 were again used as representative markers for PSI, PSII, and LHCII, respectively. In low light (150 μE/m^2^/s)-grown spinach plants, we observed that the relative amount of PSI, detected as PsaD, was low in young leaves but increased steadily with leaf age. In contrast, in high light (600 μE/m^2^/s)-grown plants, the relative amount of PSI is already elevated in young leaves, and increases only slightly with age in both low light- and high light-grown plants, however, we observed that the relative amount of PSII (PsbA) decreased with age, while LHCII increased. We also observed that PSII declined more dramatically in high light-grown plants when compared to low light-grown plants (Fig. 3d). We interpret these results to mean that PSI and LHCII are relatively stable as leaves age, whereas PSII declines in conjunction with loss of photosynthetic capacity, as has been previously reported (Prakash et al. 2001a, b; Nath et al. 2013). Additionally, our results showing that there is less LHCII in young leaves of high light-grown plants, as well as that PSII declines earlier and to a greater extent in high light than in low light, are consistent with results from other studies investigating plant adaptation to growth in high light conditions (Park et al. 1997; Baroli and Melis 1998; Kouril et al. 2013). Strikingly, we noted a correlation between PSI levels and the appearance of PSI-MC band 2, i.e., increases in PSI-MC band 2 correspond to increases in PsaD levels (i.e., PSI marker). This does not appear to be the case for PSI-MC band 1, which only appeared in old leaves under high light (Fig. 3d, lane 6). However, the amount of PSII in these leaves (old leaves of high light-grown plants) is dramatically reduced relative to younger leaves, i.e., the ratio of PSI to PSII is very high (Fig. 3d, lane 6). Taken together, we conclude from these experiments that developmental increases in PSI-MC formation resulting from age and growth light conditions correspond well with increasing amounts of PSI, and possibly specifically with an increasing ratio of PSI relative to PSII.

### Treatment of thylakoids with EDTA destroys PSI-MCs

As previously stated, we observed that the formation of PSI-MCs often occurred concurrently with the loss of the band 4 complex, and vice versa, as can be seen in Fig. 3a, b. We have also shown above that PSI-MCs appear to form when PSI levels are high and PSII levels are low. These findings suggest an involvement of PSII in the regulation of PSI-MC dynamics. We have also demonstrated that the PSI-MCs are located in the stromal lamellae (Fig. S2c). Taking these observations together, we hypothesized that granal stacking, which governs the compartmentation of the two photosystems (Pribil et al. 2014), and which is profoundly influenced by both LHCII and PSII (Herbstova et al. 2012), could be involved in PSI-MC formation.

**Fig. 4:**
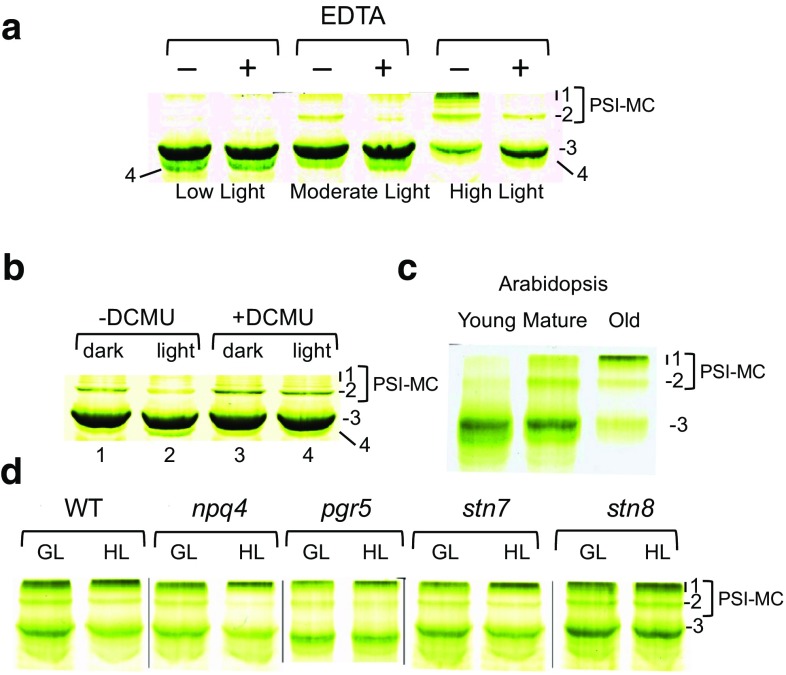
Photosystem I megacomplexes require thylakoid stacking and are induced by signaling through Linear Electron Flow (LEF). **a** Thylakoid unstacking by EDTA treatment. Spinach plants were grown under low light (150 µE/m^2^/s), moderate light (300 µE/m^2^/s), or high light (600 µE/m^2^/s). Thylakoids extracted from mature leaves were incubated on ice for 10 min either in standard TMK buffer containing 50 mM Tris–HCl, 10 mM MgCl_2_, 10 mM KCl, [− EDTA] ,or in 50 mM Tris buffer containing 30 mM KCl and 10 mM EDTA [+ EDTA]. Thylakoids were recovered, solubilized ,and resolved by green gel analysis. **b** Blocking of LEF by DCMU treatment. Spinach seedlings grown on vermiculite were harvested with roots intact and incubated overnight in water with (+) or without (−) DCMU (40 µg/mL). Cotyledons were harvested in the ‘dark’ before the lights (150 µE/m^2^/s) were turned on or 30 min after the ‘light’ were turned on. Thylakoids were extracted with DM/OG and complexes were analyzed by green gel PAGE. **c** Leaf age series of high light-grown Arabidopsis plants. Arabidopsis was grown under 600 µE/m^2^/s illumination for 6 weeks. Leaves were grouped as emerging/unexpanded (Young), first fully expanded (Mature), and last fully expanded (Old). Thylakoids from each leaf age were extracted with DM/OG and complexes were analyzed by green gel PAGE. **d** Induction of PSI-MCs by high light treatment of various Arabidopsis photosynthetic mutants. Arabidopsis plants grown under 150 µE/m^2^/s illumination for 6 weeks were transferred to 600 µE/m^2^/s for 2 days and then returned to 150 µE/m^2^/s illumination. Mature leaves were then sampled midday under 150 µE/m^2^/s growth light (GL) conditions or after an additional 30 min exposure to 600 µE/m^2^/s of high light (HL) treatment. *WT* wild type, *npq4* non-photochemical quenching 4, *pgr5* proton gradient regulation 5, *stn7* State Transition 7, *stn8* State Transition 8. PSI-MCs were defined as previously described in Fig. 1

To test this hypothesis, we first performed an artificial (non-physiological) grana-unstacking experiment. Forced unstacking of the grana is known to cause the random intermixing of the two photosystems (van der Weij-de Wit et al. 2007) and might therefore be expected to have a strong impact on PSI-MC formation and stability. It has been shown that magnesium ions are necessary for granal stacking and stability (Arntzen and Ditto 1976). The addition of EDTA to thylakoids sequesters magnesium ions and results in the rapid loss of the granal stacks (Chow et al. 1980). In Fig. 4a, spinach plants were exposed to increasing light intensities to initiate varying degrees of PSI-MC induction. Thylakoids were then isolated from these plants, treated with or without EDTA for 10 min, solubilized in DM/OG and then subjected to green gel analysis. As shown in Fig. 4a, treatment of spinach thylakoids with EDTA resulted in the selective loss of PSI-MCs that had formed in response to high light treatment. Specifically, under low light conditions there is essentially no PSI-MC induction and EDTA treatment does not appear to have any effect on the main PSI band (band 3) or the band 4 complex (band 4). However, after moderate light treatment some PSI-MC formation is visible and EDTA treatment resulted in a loss of PSI-MC with a concomitant increase in band 4 (Fig. 4a). In high light-treated leaves, there is a strong PSI-MC induction and these complexes are almost completely lost after treatment with EDTA (Fig. 4a). These results suggest that the integrity of the granal stacking is necessary for maintenance of PSI-MCs. However, it should also be noted that it is possible that magnesium chelation may also directly interfere with protein–protein interactions, e.g., with interactions between LHCII proteins, which are known to involve magnesium ions. Further experiments will therefore need to be conducted to conclusively demonstrate the direct necessity of granal stacking in PSI-LHCII MC maintenance.

### Linear electron flow and PSII phosphorylation are involved in PSI-MC induction

The EDTA-induced unstacking of grana, as shown in Fig. 4a, is a relatively non-physiological means of altering grana dynamics and thus altering PSI-MC stability. As we have also pointed out, magnesium chelation may also have other consequences for protein–protein interactions. Alternatively, grana stacking can be manipulated under more physiological conditions by merely changing light conditions. For example, it has been shown that grana stacks expand in diameter in darkness, while under high light conditions grana margins swell and the grana core contracts (Pribil et al. 2014). These dramatic changes in grana structure are attributed to phosphorylation of the LHCII antenna and the PSII core proteins by the Stn7 and Stn8 kinases, respectively (Tikkanen et al. 2008a, b; Fristedt et al. 2009; Pribil et al. 2014). Both the Stn7 and Stn8 kinases are activated by photosynthetic Linear Electron Flow (LEF), which thereby connects kinase activity to changes in the light environment. We have previously shown that PSI-MCs are regulated by changing light conditions, i.e., they are lost upon transition from dark to growth light and induced by high light (Fig. 3a, b). We have also demonstrated that loss of granal stacking appears to result in the loss of PSI-MCs (Fig. 4a). Based on these findings, we therefore hypothesized that the observed dynamics of PSI-MC formation in response to various light conditions could be a function of LEF and PSII/LHCII phosphorylation.

To investigate this hypothesis, we first tested the dependence of PSI-MCs induction on LEF. For this experiment, the inhibitor DCMU was used to block LEF at PSII (Fig. 4b). Seedling spinach plants were harvested with their roots intact and immersed in DCMU-saturated water overnight in the dark. The effectiveness of this treatment was monitored by chlorophyll fluorescence imaging. In Fig. S5, a representative chlorophyll fluorescence image showing Φ PSII in treated and untreated Arabidopsis plants reveals complete inhibition of electron transport through photosystem II. In our experience, complete inhibition of photosystem II was often achieved within one hour of the beginning of treatment and treated plants never recovered photosystem II activity.

For this experiment, DCMU-treated and untreated plants that had been kept in the dark overnight were then either exposed to normal growth light conditions for 30 min or kept in the dark. In untreated plants, exposure to growth light resulted in the dismantling of PSI-MC and formation of the band 4 complex, as expected (Fig. 4b). In DCMU-treated plants, however, exposure to growth light had no effect (Fig. 4b). From this experiment, we conclude that signaling through LEF plays a role in regulating PSI-MC dynamics and is necessary for light-induced dismantling of PSI-MCs.

To test for the involvement of specific signaling pathways downstream of electron flow from PSII it was necessary to switch experimental systems, from spinach plants to *Arabidopsis*, in order to take advantage of the available mutant lines. Initially, we had observed that *Arabidopsis* plants have little or no PSI-MCs under standard growing conditions (Fig. 1). However, based on our observations with spinach, we found that *Arabidopsis* plants grown under high light conditions (600 µE/m^2^/s), while still showing no PSI-MC in young leaves, did develop PSI-MCs as the leaves aged (Fig. 4c). In addition, to verify that these apparent PSI-MC bands in *Arabidopsis* correspond to the PSI-MCs described here for Spinach, we compared the *Arabidopsis* and spinach PSI-MC bands directly by native green gel electrophoresis and by silver-stained 2D-SDS-PAGE (Fig. S6). Figure S6a shows a side-by-side comparison of PSI-MC bands induced in both spinach and *Arabidopsis* with PSI-MC bands 1 and 2 migrating essentially in the same positions on the gel for both species. *Arabidopsis* PSI-MC bands were further verified to contain photosystem I megacomplexes in Fig. S6b, by comparison of the two-dimensional banding pattern of these complexes with spinach PSI-MCs. In Fig. S6b, which shows the pertinent gel region containing small subunit bands characteristic of photosystem I, it can be seen that the *Arabidopsis* megacomplex bands are indeed photosystem I complexes, sharing the same subunit composition as the main photosystem I complex in the same fashion as the spinach PSI-MCs. We conclude that the high light-inducible megacomplex bands in *Arabidopsis* are comparable to the PSI-MCs in spinach.

Taking advantage of the ability to induce PSI-MCs in *Arabidopsis*, wild-type (WT) and mutant *Arabidopsis* plants were treated at 6 weeks of age with temporary high light stress (600 µE/m^2^/s) for 2 days, after which they were returned to normal growth light (GL) conditions. This light treatment protocol was found to give the most dynamic PSI-MC response and resulted in a baseline induction of PSI-MCs (as defined by an increase in PSI-MC band 1 intensity) prior to these plants being re-exposed to an additional high light (HL) treatment for 30 min.

*Arabidopsis* mutant lines which are affected downstream of PSII, i.e., downstream of the effects of DCMU treatment, were selected for testing. While phosphorylation through the Stn7 and Stn8 kinases was suspected to be responsible for PSI-MC dynamics, there are other major signaling pathways downstream of PSII that could also be involved. In addition to the *stn7* and *stn8* mutants, which are defective in their respective kinases, the *npq4* and *pgr5* mutants were also included for comparison in our analysis. These two mutants are defective in aspects of photosynthetic signaling through the trans-thylakoid pH gradient. Both LEF and cyclic electron flow (CEF) contribute to the production of the trans-thylakoid pH gradient and acidification of the thylakoid lumen. Acidification of the lumen in turn causes the protonation of the PsbS protein, the Npq4 gene product, which results in the activation of the qE component of non-photochemical quenching (NPQ) (Li et al. 2000, 2002). The *npq4* mutant lacks the PsbS protein and is defective in qE. The *pgr5* mutant is defective in CEF contributing to acidification of the lumen, thereby resulting in an under-induction of NPQ and chronic over-reduction of the plastoquinone pool (Munekage et al. 2002).

Representative green gels for WT and the various mutants described above are shown in Fig. 4d. Under growth light (GL) conditions, both WT and various mutants tested already had significant levels of PSI-MC bands present, since all of these plants had been exposed to a high light treatment prior to being returned to baseline GL conditions. However, after a subsequent 30 min HL treatment, various levels of further induction of PSI-MC could be observed in WT and certain mutant plants. In this experiment, PSI-MC induction was defined as a decrease in the density of PSI-MC bands 2 and/or band 3, along with a concomitant increase in the density of PSI-MC band 1 (Fig. 4d). Based on these criteria, *npq4, pgr5*, and *stn7* mutants all appeared to show an induction of PSI-MC band 1, along with a simultaneous loss of PSI-MC band 2 after HL treatment similar to that observed for WT plants, although in the *npq4* mutant the induction appeared more pronounced. Surprisingly, the *stn8* mutant displayed the strongest phenotype, having an initial high level of PSI-MC at GL conditions but then showing minimal changes in overall PSI-MC band levels after HL treatment. PSII core phosphorylation therefore appears to be of particular importance to the dynamics of PSI-MCs.

Based on the above observations in Arabidopsis mutants, we then looked at the degree of phosphorylation of thylakoid proteins from our green gel bands. As shown in Fig. S7a, the very high amount of phosphorylated D1 protein in band 4 is immediately noticeable. However, since these samples were electroeluted from gel bands and normalized based on total protein, rather than on the amount of D1 protein per se, it is not clear from this figure whether the D1 protein in band 4 is specifically more phosphorylated than in the PSII core band. In order to make this comparison, samples from band 4 and the PSII core band were normalized based on Western blotting for D1. A titration based on equal loading of D1 protein was performed for each of the two bands, followed by blotting for phospho-serine/threonine. The results of this titration are shown in Fig. S7b. Phosphorylation of D1 in band 4 can clearly be seen to be more than sixfold higher than that in the PSII core complex. We conclude that the band 4 complex contains highly phosphorylated PSII, which is consistent with our above findings of the involvement of band 4 in PSI-MC dynamics (Fig. 3), and of the necessity of the STN8 kinase for these dynamics (Fig. 4).

### Inhibition of PSII turnover induces PSI-MCs

We have previously shown in Fig. 3c that PSI-MCs are most prevalent in old age, when PSII is actively being degraded and the ratio of PSI to PSII is highest. The involvement of Stn8 signaling in PSI-MC induction, as described above, further suggests a role for PSI-MC in PSII turnover, since PSII core phosphorylation has been shown to be involved in the PSII repair cycle (Tikkanen et al. 2008a, b). To test for the involvement of PSI-MCs in PSII turnover, we therefore asked whether PSI-MCs could be induced by artificially depleting PSII with lincomycin treatment. In the presence of lincomycin, which inhibits chloroplasts protein synthesis, it has been shown that the D1 protein is rapidly degraded due to the high rate of turnover of the D1 protein relative to other chloroplast proteins (Pokorska et al. 2009). As seen in Fig. S8, seedling spinach plants treated with lincomycin lost band 4 and gained PSI-MC band 2 and a small amount of band 1. Preventing the synthesis of new PSII therefore appears to be sufficient to induce PSI-MC formation (Fig. S8). Notably, the specific loss of the band 4 complex, concomitant with induction of PSI-MC by lincomycin treatment, again demonstrates the interdependency of these two complexes.

### Photosystem I subunits PsaL and PsaH increase as leaves age

It is not clear whether PSI-MC accumulation in older leaves is specifically a result of rising PSI/PSII ratios, as is implied by our experiments in Fig. 3, or whether other factors may also be contributing. Recent findings by Seok et al. (2014) have shown that the PsaL/H cluster within the PSI may play a critical role in modulating PSI complex formation under stress conditions, e.g., they have shown that stabilization of PsaL yields enhanced drought and photosynthetic stress tolerance (Seok et al. 2014). Similarly, we have shown that PSI-MCs form in response to photosynthetic stress (i.e., high light treatment, see Fig. 3). It has also been shown that the PsaL/H cluster forms the docking site for LHCII, thus allowing for the formation of PSI-LHCII supercomplexes (Lunde et al. 2000; Mazor et al. 2015), while we have also found that LHCII is enriched in both PSI-MCs, and especially PSI-MC band 1 (Fig. 2a). From these observations, we hypothesized that the PsaL/H cluster may play a role in PSI-MC accumulation as leaves age.

**Fig. 5:**
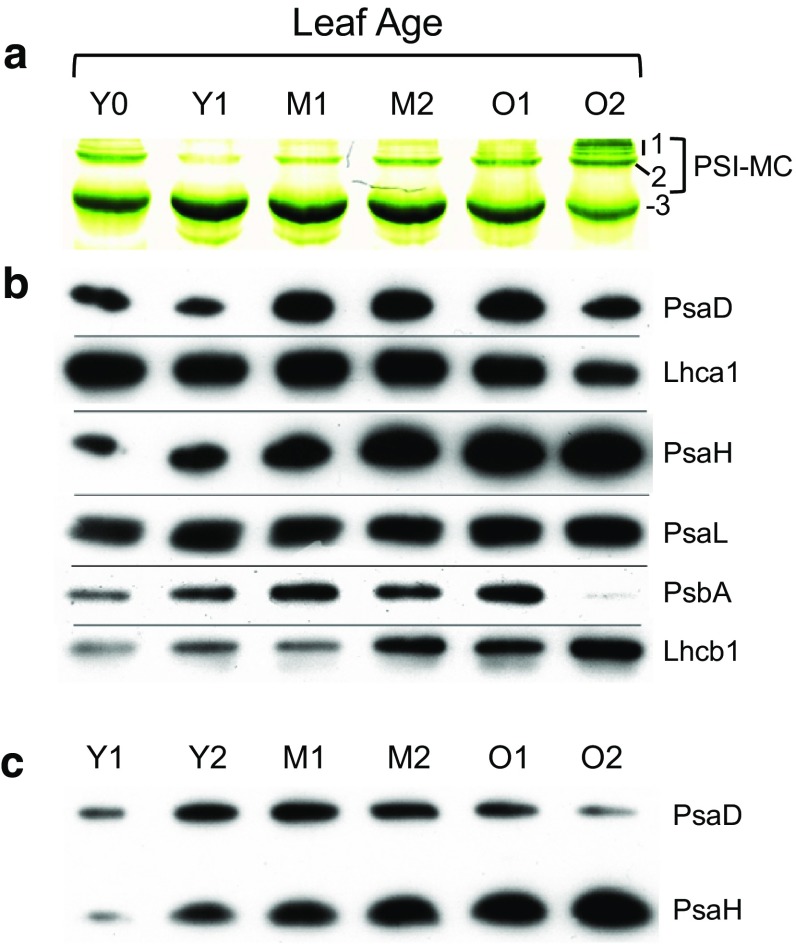
Non-stoichiometric changes in photosystem I subunits as leaf ages. **a** Photosystem I-MCs trend in leaf age series. Leaves of 6-week-old spinach plants were grouped by age from youngest to oldest as young (Y), mature (M), and old (O): yellow unexpanded leaves (Y0), first set of expanding leaves (Y1), second set of expanding leaves (Y2), first set of fully expanded leaves (M1), second set of fully expanded leaves (M2), second-to-last set of fully expanded leaves (O1), and last set of fully expanded leaves (O2). Leaves were sampled midday. **b** Western blotting for major photosystem components. The same samples that were loaded onto the green gel in panel A were denatured and run on SDS-PAGE, followed by Western blotting. **c** Simultaneous Western blot of PsaD and PsaH from leaf age series. A leaf age series from a different set of spinach leaves than the one used for panels A and B is shown. The membrane was probed for both PsaD and PsaH simultaneously to clearly show the changing ratios of the two proteins relative to one another

To investigate this possibility, a more complete leaf age series was collected to look more closely at differences in photosynthetic components over the life of a leaf (Fig. 5). Spinach plants at the age of 6 weeks were used in order to provide a spread of leaf ages that ranged from young to visibly yellowing older leaves. For this analysis, leaves were numbered starting from the youngest part of the plant at the center of the rosette (Fig. S9a). Unexpanded, greening leaves were designated Y0, the first and second expanding leaves were designated Y1 and Y2, respectively, the first and second fully expanded leaves were designated M1 and M2, respectively, and the second-to-last and last adult leaves were designated O1 and O2, respectively (Fig. S9a).

The leaf age series described above was characterized for PSI-MC formation by green gel electrophoresis and by Western blotting for markers of photosystem components (Fig. 5a, b). In addition, chlorophyll *a*/*b* ratio measurements and 77K chlorophyll fluorescence analysis were also performed on this leaf age series (Figs. S9b, c). Surprisingly, Y0 leaves were found to have high levels of PSI-MC band 2 (Fig. 5a). There was a dramatic loss of PSI-MC in the next older leaves to Y0, the Y1 leaves, which have very little PSI-MC, similar to previous observations with young leaves. PSI-MC band 2 intensity increased gradually as leaves aged from Y1 through O2, with PSI-MC band 1 ultimately appearing in the visibly yellowing leaf, O2. In Fig. 5b, Western blotting of total thylakoid protein was performed from the same samples shown in Fig. 5a. The pattern of expression for PsaD (PSI marker) and Lhca1 in these samples mimics the dip and rise in PSI-MC formation seen on the green gel in shown Fig. 5a, except in older leaves (O1 and O2) where the abundance of PsaD and Lhca1 slightly decline, while green gels show PSI-MCs increasing for these samples. Hence, the overall pattern of PSI-MC formation, i.e., the sudden dip in PSI-MC formation in young leaves (Y1) followed by a gradual rise in PSI-MC formation as leaves mature, is closely reflected in the expression pattern of PSI, as indicated by PsaD and Lhca1 levels. The exception to this pattern, as we have noted, is in old leaves, where PSI-MC levels are high despite a slight decline in PSI. This discrepancy led us to look more closely at PSI subunit protein levels, as described below.

Before continuing to discuss PSI subunit stoichiometry in greater detail, we wished to expand on the unique finding of an initial drop in PSI in Y1 leaves, as indicated by blotting for PsaD (Fig. 5b). This drop is unusual, since it appears to indicate PSI degradation in young leaves. However, due to the fact that samples are normalized by total chlorophyll content, the drop in PSI could also represent synthesis of PSII and/or LHCII, which constitute the bulk of the remaining chlorophyll in the thylakoids. Western blotting for PsbA and Lhcb1 in Fig. 5b seems to indicate that this is the case since PsbA (D1 protein) levels rise in young leaves from Y0 through M1. Likewise, there is also a strong increase in Lhcb1 from Y0 to Y1. Large-scale synthesis of photosystem II and LHCII in Y1 leaves is further supported by chlorophyll *a*/*b* ratio and 77 k chlorophyll fluorescence data (Fig. S9b, c). For example, in Y1 leaves there is a dramatic drop in the chl *a*/*b* ratio that parallels both the loss of PSI-MC band 2 and the decline in PsaD (Fig. S9b; Fig. 5a, b). At the same time, there is also a decrease in the relative PSI fluorescence, with a concomitant rise in free LHCII fluorescence (Fig. S9c). It is interesting to note that between Y0 and Y1 there is also an apparent reversal of the direction of state transitions, i.e., PSI fluorescence actually decreases upon shifting from dark to light in Y0 leaves, while in Y1 leaves PSI fluorescence increases upon exposure to light, as would normally be expected for transition from state 1 to state 2 (Fig. S9c). Collectively, from these data, we conclude that there is little PSII in Y0 leaves relative to PSI, and that synthesis of both PSII and LHCII as leaves progress from Y0 to Y1 results in observed drop in the PsaD signal, since PSI then represents a smaller fraction of total chlorophyll. The same reasoning also accounts for the decline in both the chlorophyll *a*/*b* ratio and the relative PSI fluorescence for these samples.

Returning to PSI subunits stoichiometry, as visualized in Fig. 5b, we further observed that PsaH and PsaL do not conform to the same expression pattern as seen for other PSI subunits, since both components increase steadily in abundance from initial greening (Y0) through maturity (O2). Further, we observed that the ratio of PsaH versus PsaD appears to rise significantly. To confirm this observation, since these blots do not present strictly quantitative data, but merely qualitative comparisons, Fig. 5c presents a ‘simultaneous’ Western blot for both PsaH and PsaD in which the changing ratios of the two PSI components are clearly visible, and cannot be attributed to differences in development between blots. PsaH, and possibly PsaL, therefore appear to increase in abundance, relative to PsaD, as leaves age.

## Discussion

In this paper, we have described the finding of a series of very large Photosystem I-LHCII megacomplexes, which are present to varying degrees in various species. These complexes appear to be very different in composition and behavior from the classical PSI-LHCI-LHCII State Transition complex, which is normally assigned to a single band on native gels. In contrast, the complexes described here seem to represent not simply a single kind of association of LHCII with PSI, but a range of such associations with increasingly larger complements of LHCII per PSI center. These complexes, moreover, are induced in what is essentially the opposite pattern normally expected for a State 2 PSI-pLHCII complex, i.e., they are formed in the dark and in response to high light, rather than under low-to-moderate light conditions. Photosystem I preparations with unexpectedly large complements of LHCII have been described previously (Bell et al. 2015), and even specifically in the context of senescent leaves (Prakash et al. 2001a, b), as we have also described here. None of these findings have, however, described the dynamic behavior of these complexes or attempted to elucidate their function in the context of a physiological photosynthetic process.

A strikingly consistent feature of the PSI-MC behavior that we have observed and characterized here is that they are increasingly formed in response to light and aging stress (see Figs. 3, 5), and that these two factors can act independently and additively to contribute to the amount of PSI-MC that is made. This is reasonable from a physiological standpoint, inasmuch as high light and aging are interrelated stressors, i.e., high light stress causes accelerated senescence and aging exacerbates high light stress (Khanna-Chopra 2012). In this way, it seems that PSI-MCs are an adaptive mechanism in response to cumulative light stress. That being the case, how then are these complexes formed and what function in adapting to high light, or perhaps resulting from high light, do they serve?

**Fig. 6:**
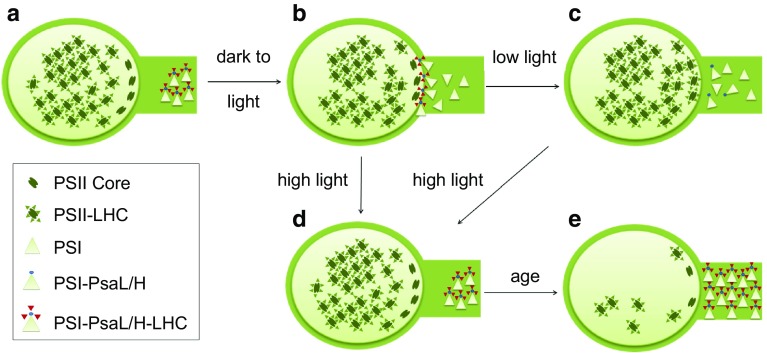
Model of PSI-MC dynamics. **a** After overnight darkness PSI-MCs in the stromal lamellae are associated with a portion of the mobile LHC pool. **b** At the onset of daylight, PSI-MCs associate with PSII at the grana margins, forming the band 4 heterocomplex. **c** In low light, PSI-MCs are dismantled and the LHC associated with the PSI-MCs becomes part of the PSII antenna in the grana. **d** In high light, LHC remains associated with PSI-MCs, leaving PSII cores exposed at the grana margins and facilitating PSII turnover. **e** Increasing expression of PsaL/H with age allows more of the LHC pool to associate with PSI in PSI-MCs, releasing PSII to be degraded. This process is accelerated by induction of PSI-MC formation in high light

The exact signaling mechanisms by which PSI-MCs are formed and dismantled remain an open question. We do not attempt a detailed elucidation of signaling in this work, except to the extent that linear electron flow and PSII core phosphorylation were found to be involved. Our data indicate that PSII phosphorylation by the Stn8 kinase is important for induction of PSI-MC, since lack of PSII phosphorylation resulted in an inability to induce PSI-MCs after high light treatment (see Fig. 4d, *stn8* mutant). This observation is revealing, in that PSII phosphorylation has also been specifically linked to PSII turnover, including dismantling of PSII-LHCII supercomplexes, mobility of PSII core complexes to the grana edges, and an increase in the relative amount of the grana edge regions through shrinking of the grana core and “breathing” of the edges of the granal stacks (Puthiyaveetil et al. 2014; Tikkanen et al. 2008a, b). The overall effect of PSII phosphorylation therefore involves the increased exposure of the PSII core at the edges of the granal stacks. We have shown that the band 4 complex contains highly phosphorylated PSII (Fig. S7b), and many of our results show that there is an apparent tradeoff between the formation of PSI-MC and the formation of the band 4 complex (e.g., see Figs. 3a, b, 4a, b, and Fig. S7). From these data it seems reasonable to infer that the band 4 complex represents a possible interaction between Photosystem II and Photosystem I where the grana and stromal lamellae meet. We believe that the possible interaction of PSI with PSII at the edges of the granal stacks may play a crucial role in PSI-MC dynamics, as described further in a model presented below (Fig. 6).

Whereas PSII core phosphorylation appears to be involved in PSI-MC dynamics, the ability to form PSI-MCs seems to depend on PSI itself. We have shown that the strength of PSI-MC induction correlates both with increasing relative abundance of PSI (see Figs. 3d, 5b), and increasing expression of PsaH/L (see Fig. 5b, c). We have also demonstrated that the PSI-MCs are more likely to have a full complement of PsaL and PsaH. As has been previously noted, the PsaH/L cluster forms the docking site for LHCII in higher plants, and so this is consistent with our finding that PSI-MCs represent higher-order PSI-LHCII complexes (see Fig. 2). While this variability in PsaH and PsaL strongly suggests to us a regulatory function for these subunits, it is nevertheless unclear from our findings whether they play a causative role in PSI-MC formation. It is not clear, for instance, whether PSI-MCs form because they contain PsaH/L or whether the main PSI complex appears not to contain PsaH/L simply because they were lost during solubilization. This area appears to be deserving of further investigation.

Based on the aspects of PSI-MC behavior described above, we propose a model of PSI-MC function, described in Fig. 6. At its heart, this model simply involves the association of PSI in the stromal lamellae with PSII-LHCII complexes at the edges of the grana where the two compartments meet, which is not dissimilar, for example, from the model presented in Tikannen et al. (2008a, b). In our model, however, we contend that this PSI-PSII-LHCII complex, which we believe may be the band 4 complex, as designated in this work, exists as a dynamic intermediate between states, rather than serving as an end in itself. The formation of this heterocomplex at the grana margins is necessary for the transfer of LHCII between the two photosystems, and therefore for the assembly and disassembly of PSI-MCs. Association of PSI with PSII-LHCII allows the handoff of LHCII from PSII to PSI while, conversely, recruitment of PSI-MCs to the grana margins allows donation of LHCII back to PSII. Presumably, both processes operate in dynamic equilibrium. The direct handoff of LHCII back and forth between the two photosystems eliminates the problem of ‘free’ LHCII, i.e., it is not clear how LHCII migrates independently from the granal stacks to the stromal lamellae, and does so without producing fluorescence and without resulting in significant photo-oxidative damage. This interpretation places a renewed emphasis on the structure of the thylakoids as a way to regulate the interaction of the two photosystems with one another.

Historically, the movement of LHCII has been understood as a mechanism for balancing light absorption between the two photosystems. More recently, the description of complexes in which PSI associates directly with PSII at the grana margins has also been interpreted in this context, as a way to balance excitation pressure, albeit in the absence of classic state transitions (Mekala et al. 2015). The behavior of the complexes that we have described in this paper does not seem to us to be well explained by this model, i.e., it does not seem likely to us that the primary purpose of these megacomplex dynamics is to balance excitation pressure. Rather, as stated, the evidence presented in this paper seems to point to a role for PSI in excitation quenching and in PSII turnover. In this way, it is possible to imagine that a mechanism may be provided for activity of the Stn7 and Stn8 kinases to set the rate of PSII turnover. Testing the various aspects of this model, deciphering the detailed signaling mechanisms involved, and placing these within the context of current models of photosynthetic processes will provide ample subject matter for future work.

## Conclusions

We have demonstrated the existence of energetically coupled PSI-LHCII complexes in the stromal lamellae consisting of multiple LHCII trimers per PSI photocenters. These complexes are formed in response to high light and aging stress and require granal stacking to maintain their integrity. The accumulation of PSI-LHCII complexes with age is correlated with increasing PSI/PSII ratios and appears to involve increasing expression and relative abundance of the minor PSI subunits PsaL and PsaH. The rapid, dynamic reorganization of PSI-LHCII complexes in response to changing light intensities involves PSII core phosphorylation, and appears to involve the formation of a PSI-PSII photocenter complex that is heavily phosphorylated on PSII.

## Electronic supplementary material

Below is the link to the electronic supplementary material.

Supplementary material 1 (PDF 21769 KB)

## Abbreviations

PSI-LHC: Photosystem I light-harvesting complex
PSI-MC: Photosystem I megacomplex
PSII: Photosystem II
DM/OG: Decyl maltoside/octyl glucoside
TCSPC: Time-correlated single photon counting
