# Supplementary material for: Photosystem I-LHCII megacomplexes respond to high light and aging in plants
Source: Photosynth Res. 2017 Oct 3;136(1):107–24. doi: 10.1007/s11120-017-0447-y (PMC5851685; doi:10.1007/s11120-017-0447-y)
Supplement: Supplementary file 1 — Supplementary material 1 (PDF 21769 KB) [file 11120_2017_447_MOESM1_ESM.pdf]

Supplemental Information

Article title:

Specialized Photosystem I Complexes Respond to High Light and Ageing in Plants

Journal Name:

Photosynthesis Research

Authors:

Eliezer M. Schwarz[1*], Stephanie Tietz[1], and John E. Froehlich[1,2]

Affiliations and Addresses of Authors:

[1]Michigan State University-Department of Energy Plant Research laboratory.

[2]Michigan State University-Department of Biochemistry and Molecular Biology.

East Lansing, MI 48824, USA

Corresponding author:

Eliezer M. Schwarz e-mail: Schwa337@msu.edu

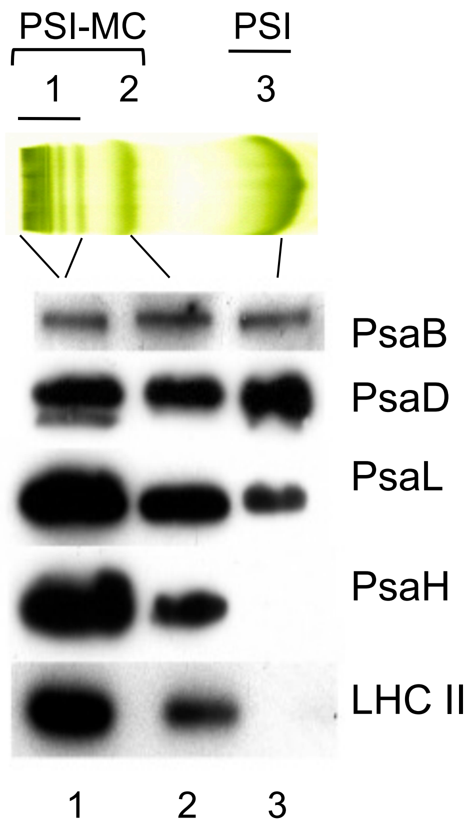

Fig. S1 Photosystem I megacomplexes are enriched in PsaH and PsaL relative to the main PSI band. Western blotting of PSI megacomplex bands was performed for additional Photosystem I subunits and for the main light harvesting antenna protein, LHCII. PSI-MC bands were excised from green gels and proteins were electroeluted from the gel slices to allow for quantitative comparison of proteins between megacomplexes. Samples were loaded to give an equivalent signal for PsaD.

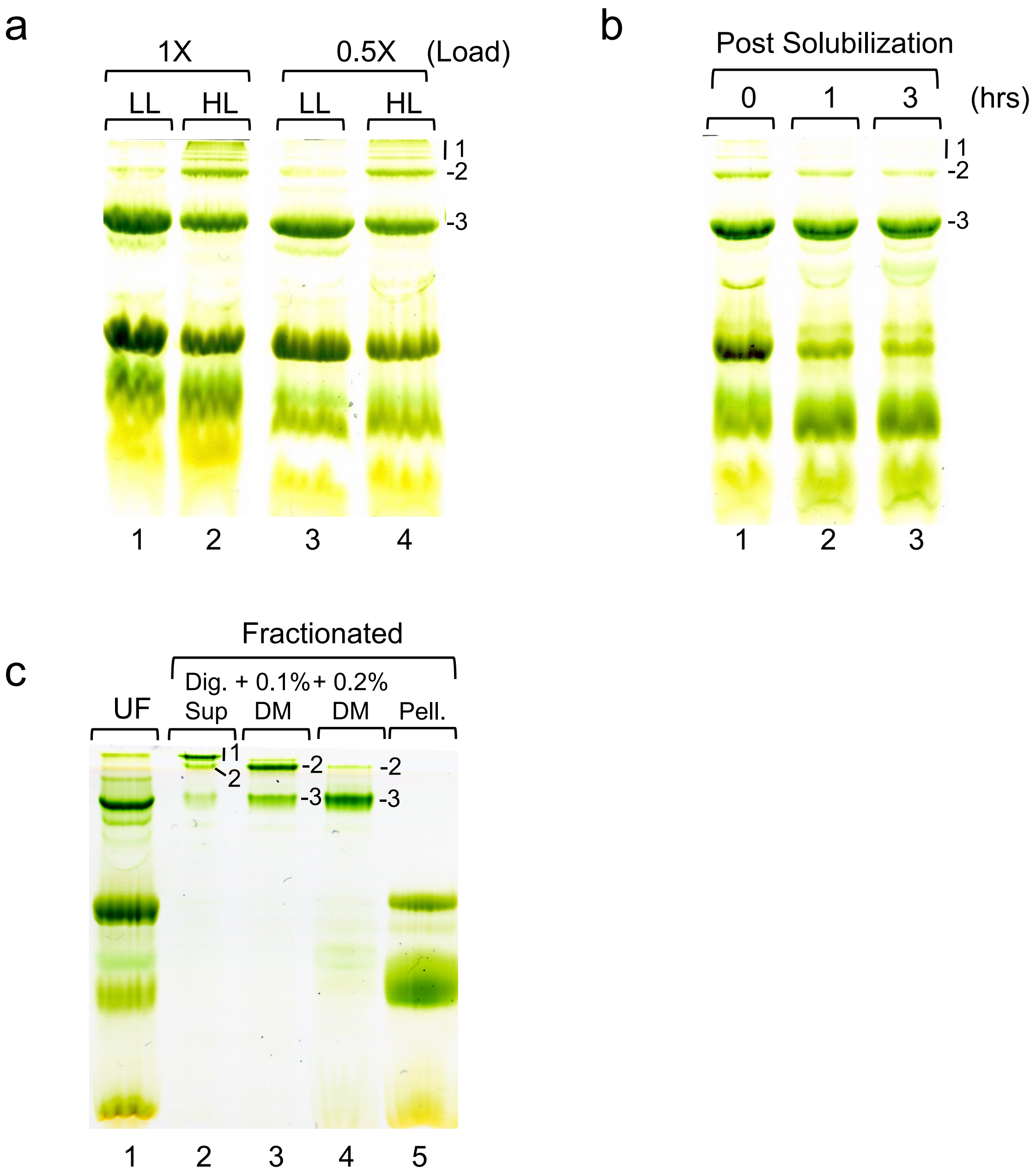

Fig. S2 PSI-MCs are non-artefactual complexes located in the stromal lamellae. **a,** High light induction of PSI-MCs is not an artefact of undersolubilization. Spinach leaves were subjected to

either to growth light (LL) or high light treatment minutes (HL) and thylakoids were either solubilized normally (1X) or at half the normal total amount of chlorophyll in the same volume of solubilization buffer (0.5X). **b,** PSI-MCs do not form as a post-solubilization aggregation effect. Spinach leaves were subjected to a mild high light treatment to induce moderate levels of PSI-MC formation and solubilized normally. Solubilized thylakoids were then allowed to remain on the bench at room temperature for the indicated time period. **c,** PSI-MCs are PSI-LHCII complexes located in the stromal lamellae. Spinach thylakoids were solubilized in 1% digitonin and the insoluble grana were pelleted by centrifugation at 15,000G for 10 minutes. The supernatant (sup) was loaded directly onto a native gel or was first supplemented with the indicated concentration of decyl maltoside (DM). The insoluble pellet (pell.) containing the grana was solubilized normally for green gel analysis.

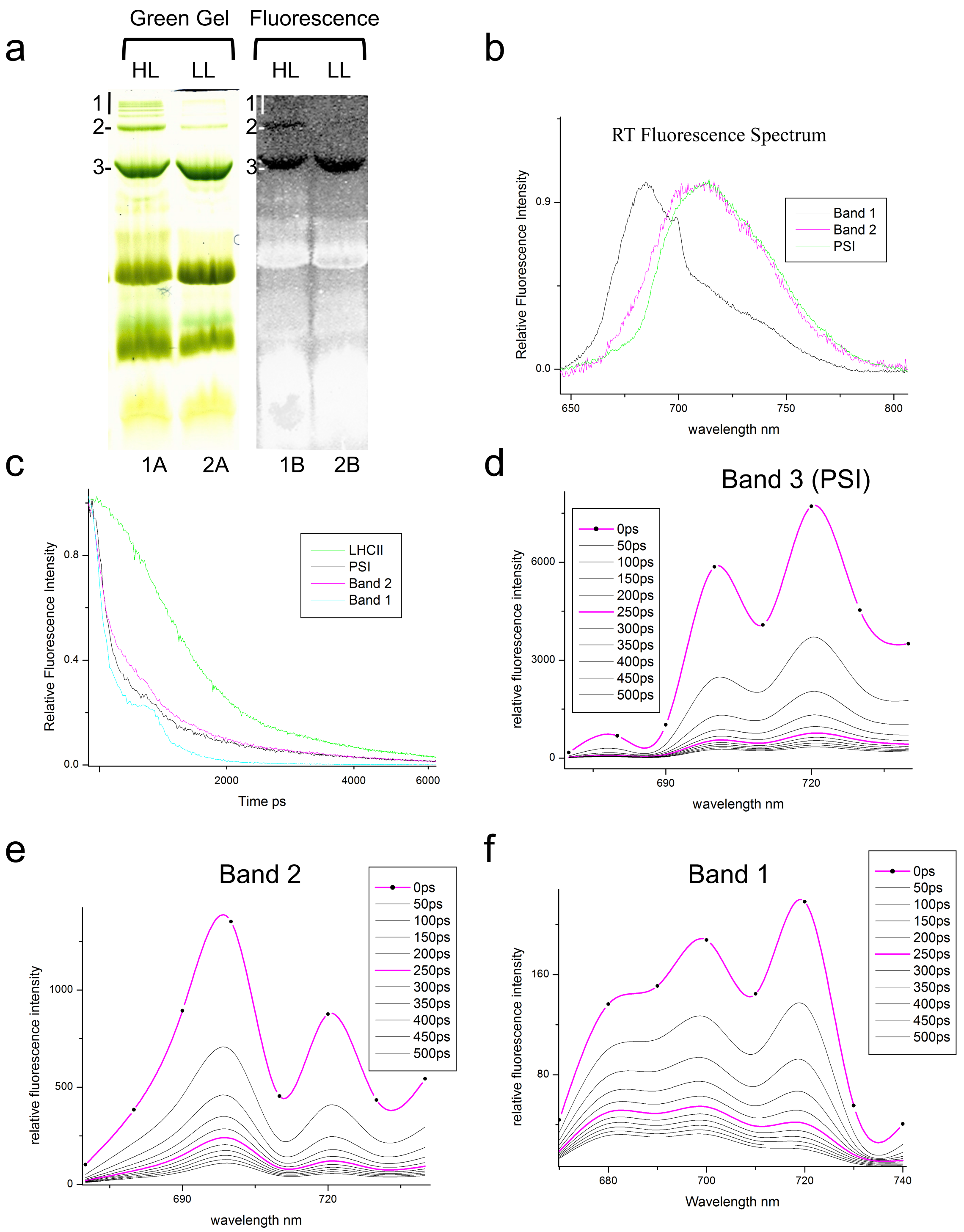

Fig. S3 LHCII is energetically coupled to PSI in PSI-MCs. **a,** PSI-MC fluorescence is strongly quenched. Spinach leaves subjected to either high light treatment (HL) or normal growth light (LL) were analyzed by green gel native PAGE and visualized on a standard long wave UV

transillusminator (Fluorescence). **b,** Room temperature fluorescence spectra of PSI and PSI-MC bands. The indicated bands were excised from green gels and excited by a 435nm LED. Spectra were normalized to their peaks in the 650-800nm range. **c,** Representative TCSPC traces at 680nm for PSI-MC bands 1 and 2, PSI band 3, and LHC trimer bands excised from green gels and excited at 435nm. Traces were normalized to their peaks. **d, e,** and **f,** Decay Associated Spectra (DAS) for the indicated green gel bands. TCSPC trances were tail-matched to their respective RT fluorescence spectra shown in **(b)** at 6ns. Data points were taken every 10nm between 670nm and 740nm, as indicated by black dots, and traces were fitted to two exponential decays by global analysis.

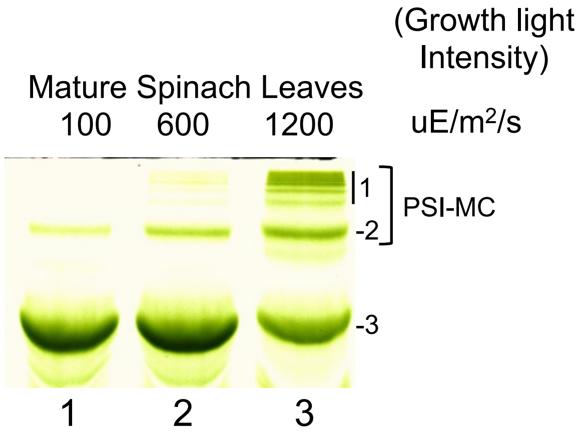

Fig. S4 PSI-MC band 1 can be induced by high light in mature leaves. Spinach plants were grown for six weeks at the indicated light intensities and mature leaves were sampled for green gel analysis.

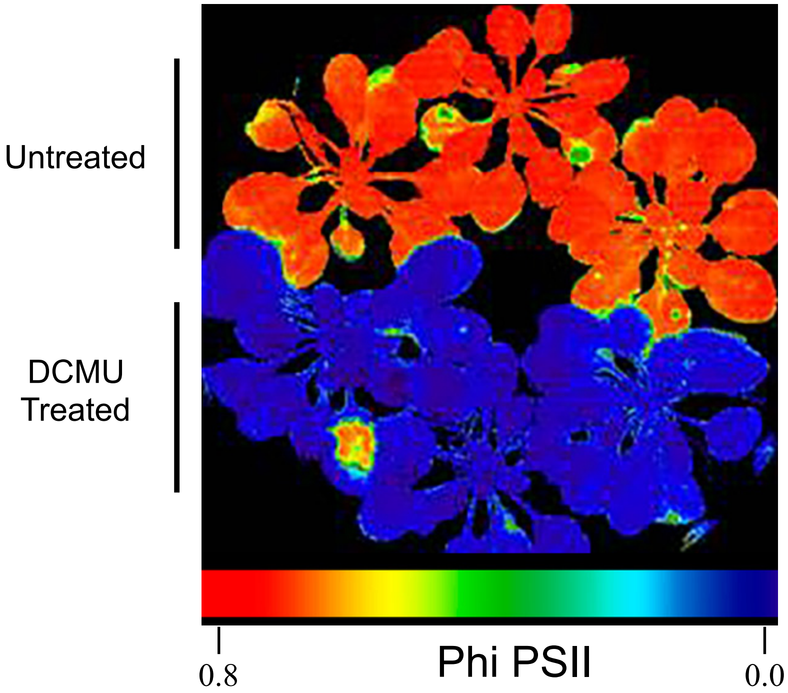

Fig. S5 DCMU treatment completely inhibits electron transport through photosystem II. False color chlorophyll fluorescence image of untreated and DCMU treated Arabidopsis plants showing Phi PSII, the photochemical efficiency of Photosystem II, where 0.8 is the maximum theoretical efficiency.

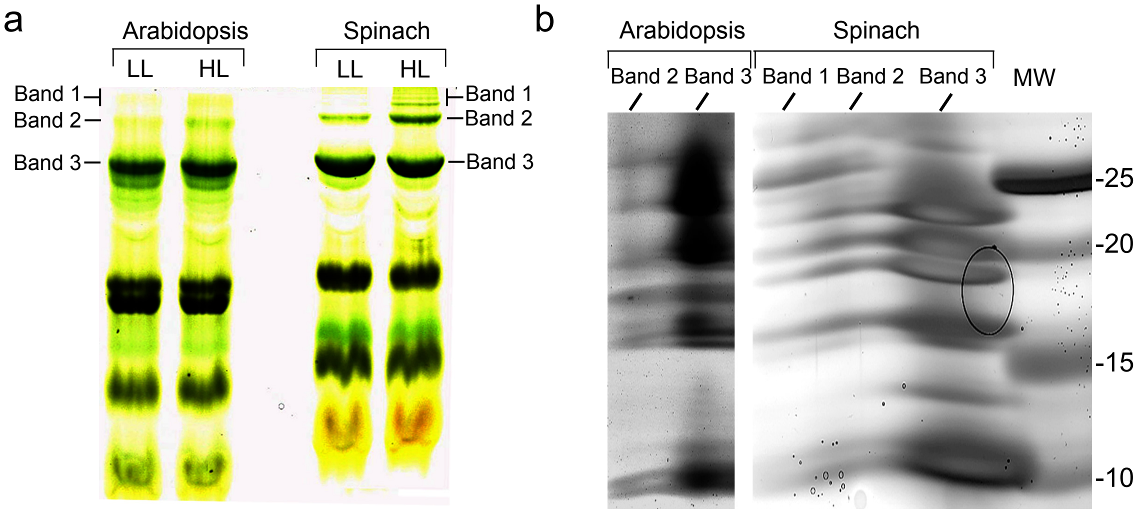

Fig. S6 PSI-LHCII-MC bands in Arabidopsis correspond to those described in Spinach. **a**, Native green gel of both Arabidopsis and Spinach showing a side by side comparison of banding patterns under low light (LL) and high light (HL) conditions. **b**, Silver stained 2D SDS-PAGE gels showing the low molecular weight component region for PSI-MC (Bands 1-2) and PSI (Band 3) from both Arabidopsis and Spinach after HL treatment.

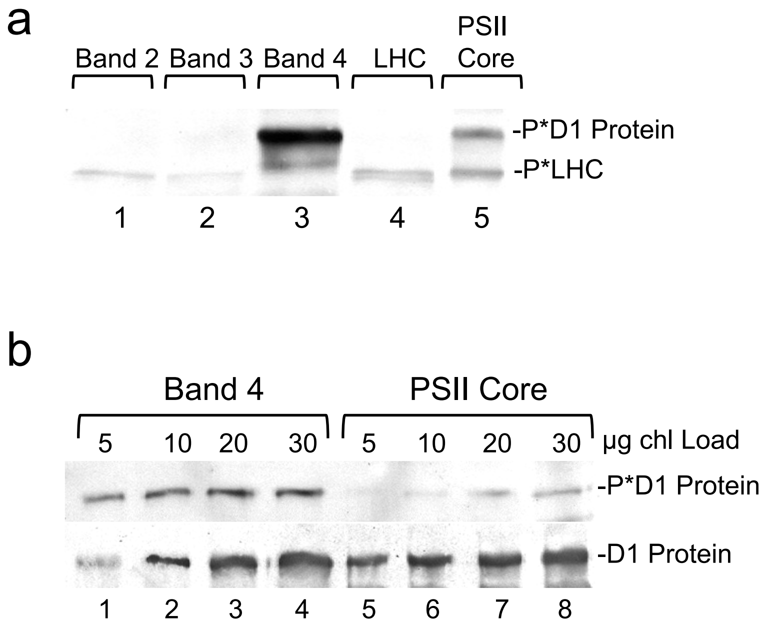

Fig. S7 The Band 4 complex contains highly phosphorylated photosystem II. **a,** Anti-phosphothreonine blot of electroeluted gel bands normalized for total protein. **b,** Quantitative comparison of D1 protein phosphorylation in the Band 4 complex versus the PSII core band. Samples were normalized based on Western blotting for D1 and then loaded as indicated. Duplicate blots were made for D1 and anti-phosphothreonine blotting.

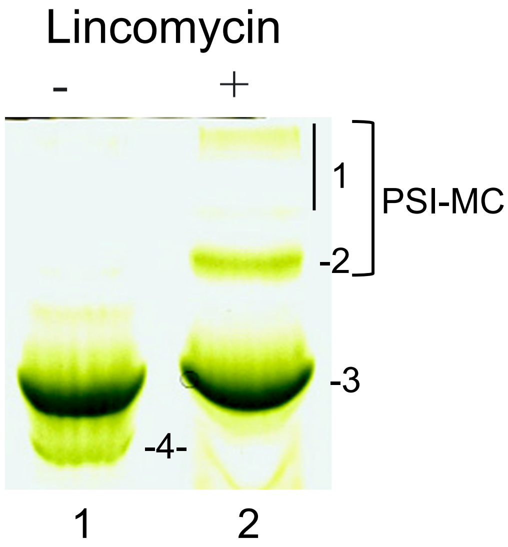

Fig. S8 Lincomycin treatment of intact spinach seedlings. Spinach seedlings were grown on vermiculite under 150 μE/m^2/s illumination and plants with fully expanded cotyledons but no true leaves were harvested with their roots intact. Seedlings were incubated with their roots submersed in water with or without lincomycin at 100μg/mL for 6 hours. Cotyledons were harvested for green gel electrophoresis. Bands 1-4 have been previously defined.

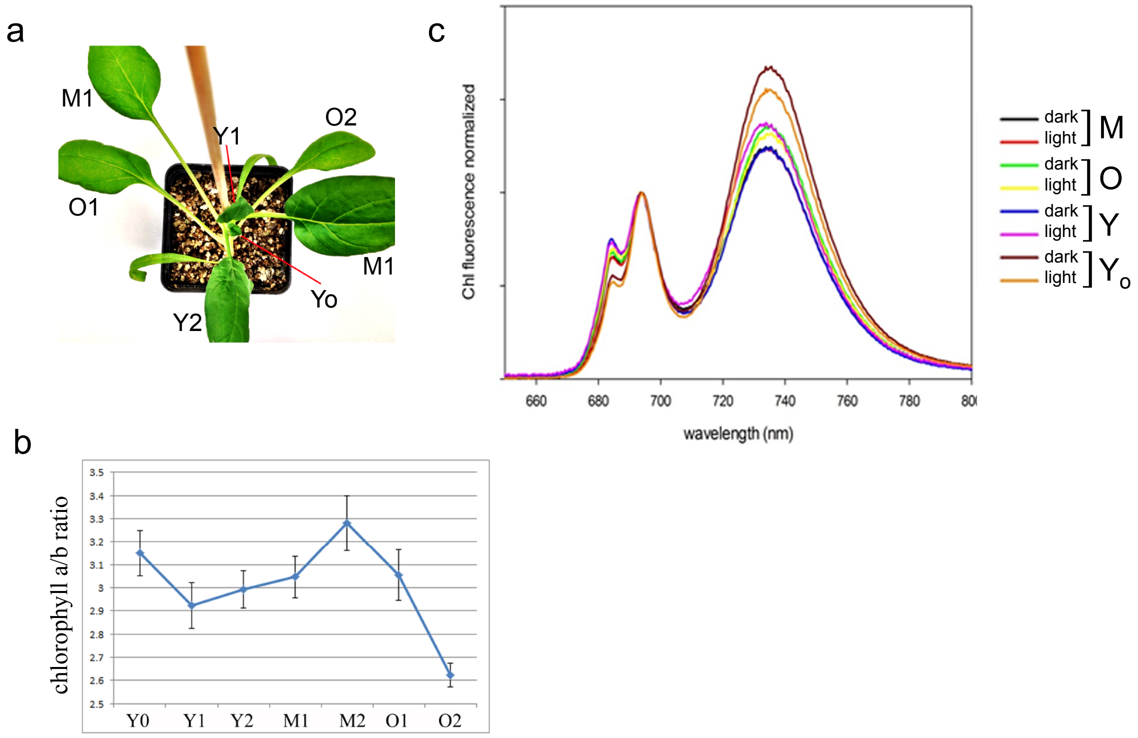

Fig. S9 Characterization of spinach leaf age series. **a,** Leaves of 6 week old spinach plants were grouped by age from youngest to oldest as young (Y), mature (M) and old (O): Yellow unexpanded leaves (Y0), First set of expanding leaves (Y1), second set of expanding leaves (Y2), first set of fully expanded leaves (M1), second set of fully expanded leaves(M2), second to last set of fully expanded leaves (O1), and last set of fully expanded leaves (O2). **b,** Chlorophyll a/b ratios for the leaf age series described in in part A. Chlorophyll was measured according to the method of Porra et al.. n=5. **c,** 77K chlorophyll fluorescence emission spectra of dark and light adapted spinach leaves from the leaf age series described in part (**a**). Spectra are representative. Multiple leaf discs were used for each curve and at least 3 biological replicates of the leaf age series were performed.
